# Understanding the effect of stay-at-home orders on psychological distress during the COVID-19 pandemic: Evidence from a longitudinal study in Australia

**DOI:** 10.1371/journal.pone.0325753

**Published:** 2025-07-02

**Authors:** Patrick Rehill, Roy Barnes, Nicholas Biddle, Ben Edwards

**Affiliations:** 1 POLIS: The Centre for Social Research, Australian National University, Canberra, Australian Capital Territory, Australia; 2 School of Politics and International Relations, Australian National University, Canberra, Australian Capital Territory, Australia; University of New South Wales, AUSTRALIA

## Abstract

The extraordinary public policy response to COVID-19 in Australia saw extended lockdowns in some Australian states and territories, some of the longest in the world. This paper seeks to understand the effect these periods of lockdown had on the mental health of those living through them. Using a staggered difference-in-differences design we study two different periods — one in 2020, one in 2021. During this time there were some jurisdictions (Victoria, New South Wales and in the second period the Australian Capital Territory) experiencing extended lockdowns while the rest of the country was largely living life as normal with no or low local transmission of the virus. While there seems to have been an effect in this first period, we do not find a statistically measurable effect in 2021 as even though distress rose during the lockdown period, it rose similarly among those jurisdictions out of lockdown as well. We speculate this may be because of adaptation to stress, growing cases in control jurisdictions, or the vaccine roll-out providing a concrete endpoint for lockdowns.

## Introduction

The COVID-19 pandemic was an occurrence without precedent in living memory and it brought with it extraordinary policy responses. Governments across the world implemented a range of policy responses to try and contain the virus [1]. Particularly severe were the stay-at-home policies pursued by many governments around the world. While many of these jurisdictions were successful in containing the spread of the virus, these policies also created concern as such a serious disruption to ordinary life could risk causing widespread psychological distress [2]. The question of whether lockdowns caused significant increases in distress and implicitly whether this distress was worth it to contain spread of the virus has been a topic of considerable debate in both the academic literature and the public sphere more broadly [3,4].

Australia had an unusual experience of the pandemic in 2020 and 2021. It was a low-infection country with case numbers that were often at or near zero, but it also had very different policies in place across the country [5]. Australia has a federal government and eight state and territory governments which collectively managed COVID-19 containment policies. For most of 2020 and 2021, the country’s international border was closed with strict hotel quarantine policies in place. These policies were largely successful in stopping the virus from spreading in from overseas [5]. When the virus did escape into the community, state and territory governments generally implemented strict containment policies including stay-at-home orders and state/territory border closures [6]. This not only served to stop the spread of the virus within the jurisdiction, but also prevented it from crossing into other jurisdictions. What this meant then is that there were times where some jurisdictions had very severe lockdowns, while in other jurisdictions (sometimes jurisdictions next-door), life was carrying on almost as normal (though with strict contact tracing and some less onerous containment policies still in place). In practice, the jurisdictions that experienced the longest lockdowns ended up being Victoria and New South Wales (NSW), Australia’s two largest states, home to its two largest cities – Melbourne and Sydney [5]. This high policy variability and low spread of the virus makes Australia an excellent place to study the effects of lockdowns on wellbeing as firstly, it means there are good quasi-experiments available and secondly, it means that the effect of the virus itself and the suffering it caused is much lower than in jurisdictions with more cases. This allows for cleaner isolation of the effect of lockdown.

In this paper we make use of eight waves of data collected roughly once every three months between April of 2020 and December of 2021 to estimate the causal effect of lockdown policies on psychological distress using a staggered difference-in-differences design [7] across two different time periods. The first time period is the wave of lockdowns that started in March of 2020 before states lifted their lockdowns over the next few months until Victoria, the last state in lockdown, finally lifted the policy in a durable way (there was a brief lifting in the winter of 2020) in October of 2020. We call this the 2020 Period. After the 2020 lockdowns, the virus seemed to be controlled through other policies. Still the international border closure was the primary line of defence, but contact tracing, capacity limits in indoor venues, and frequent testing served to identify and control the small number of cases that escaped hotel quarantine. Following October 2020 any lockdowns that did happen were generally for shorter periods to control limited outbreaks with the aim of zero community transmission [5,6].

The second major lockdown period was in mid-to-late 2021 where several states went back into lockdown to try to contain the new Delta variant of the virus. We call this the 2021 Period. This differed from the first period of lockdowns because the goal was no longer ‘COVID zero’ but rather to limit spread until the country reached widespread vaccination. There was community spread (at relatively low levels by international standards) across lockdown and non-lockdown jurisdictions and the relaxing of containment policies was tied not to transmission levels but vaccination targets. The two different periods of prolonged lockdown then are qualitatively different from each other.

There is another key difference that comes not from the reality of the pandemic but the data available. This paper uses a staggered difference-in-difference design to estimate the effect of lockdowns. This is suitable for the 2021 Period when jurisdictions went into lockdown sequentially and several jurisdictions never went into lockdown at all. However, in the 2020 Period, all the jurisdictions entered treatment at essentially the same time meaning there is no valid comparison group for measuring the effect of going into lockdown. Our solution is to study a slightly different treatment in the 2020 Period. Rather than studying the effect of going into lockdown, we study the effect of leaving lockdown (which was nicely staggered and for which we have a good comparison group in Victoria). As we would expect distress to bounce back from lockdown during this period [8], this effect of lifting lockdown can tell us something about the effect of being in lockdown.

We find some positive effect to lifting lockdowns in the 2020 Period with reductions in levels of psychological distress. The 2021 Period shows no statistically measurable average treatment effect for states in lockdown. We hypothesise that this is because psychological distress was rising across all jurisdictions in Australia at the time and that any distress from lockdowns was compensated for by rising distress in control states which were for the first time experiencing significant community spread of the virus. There is also potentially an adaptive effect at play here, where repeated lockdowns lead to less of an effect on psychological distress as people adapt to living in lockdown.

To the best of our knowledge, this is the first paper that estimates the effect of lockdowns on psychological distress in 2021 using quasi-experimental methods and representative, longitudinal data in Australia, or anywhere around the world. While much of the world abandoned long lockdowns in 2021, the Australian experience helps to understand the effect of protracted stay-at-home orders and has implications for the design of non-pharmaceutical measures to contain disease spread in future crises.

## Literature review

The effect of COVID-19 lockdowns and other containment policies on mental health and psychological distress is a widely studied topic. Many studies have been conducted around the world with a few also conducted in Australia. The effects seem to be overwhelmingly negative no matter what the population studied was [9,10]. Longitudinal studies are particularly important as they provide good evidence of within-person changes over time, allow for the easy partialing out of time invariant confounders and can demonstrate causal relationships over time [11]. To narrow down the literature to something tractable we limit ourselves to robust longitudinal, repeated cross-sectional or aggregations of studies in systematic reviews or meta-analyses. We break this down into a review of the international literature and then a look in greater depth at the few Australian studies that exist.

### The effect of COVID lockdowns on mental health

It is useful to begin by looking at the systematic reviews and meta-analyses that exist of the effects of COVID-19 on psychological distress and mental health. There are several that have been conducted. Some of the most useful for our purposes (interested in longitudinal data and generalisable findings) looked at longitudinal studies around the world [10], longitudinal studies in Britain [12], and cross-sectional studies around the world [13,14]. Of these, only Prati and Mancini [10] have an explicit focus on lockdowns (the rest look at the effect of the early pandemic overall), though all cover periods of lockdown and discuss containment policies as moderators of distress during the pandemic. In general, there is a negative effect from the pandemic or lockdowns and in particular this effect appears to be heterogeneous across and within jurisdictions. The most directly relevant meta-analysis by Prati and Mancini shows a lot of heterogeneity between samples but moderator variables like gender or age could not explain this variation. Overall, their findings suggest that most people were resilient to distress in lockdowns and that those who experienced significant increases in distress were from more vulnerable subgroups.

Most individual longitudinal studies reported negative effects from lockdowns in Europe [15–23], North America [24–28], and across a number of multi-country studies [29–31]. These studies all generally show an increase in distress when jurisdictions go into lockdown though there is some amount of heterogeneity within and between studies. It is unclear to what extent distress recovers after lockdowns are lifted. For example, Fancourt *et al*. [19] and Patel [12] showed very different results even in the same population (Britons). Different analytical approaches and ways of defining the treatment effect could explain the variation in findings (e.g. binary indicators of lockdowns versus days in lockdown and pooled OLS or a difference-in-differences model). Comparing to pre-pandemic outcomes is probably not as robust an approach [32]. The reason for this twofold, first distress was already increasing prior to the pandemic, secondly, there were other pandemic-related factors that were causing distress that were not lockdowns (e.g. travel restrictions out of state/territory/country, worry about future illness).

The research literature is also unclear to what extent extended and repeated lockdowns have a compounding effect. Grygarová *et al*. [33] see a pattern of compounding distress over time in Czechia while studies elsewhere in Europe [17,19,20] show their sample adapting to life in lockdown with distress staying stable or reducing after some period of time. What makes it difficult to compare across jurisdictions is that policies (and therefore the treatments) were quite different. Lockdowns lasted different lengths of time, the severity of the policies varied widely and what other containment policies went along with them (and are therefore not separable from lockdowns when examining the effect of treatment) varies as well [1].

A common pattern of findings is that those who were most vulnerable to the effects of the pandemic seem to have suffered most. Working parents – particularly working mothers – were hit harder in many studies due to the demands of working from home while caring for children; often while having to supervise online learning [18,21,34]. Women in general saw worse mental health effects presumably for similar reasons [15,21,29,31,34]. Financial insecurity was an important moderator as well with poorer people and those who lost their jobs showing larger negative effects across these studies [18,26]. Those living in smaller dwellings saw worse effects too, presumably due to living space at home being particularly important when one cannot leave the home [21].

There appear to be age differences as well with younger people being more distressed by lockdowns than older people. However, this is not consistent across all studies. Losada-Baltar *et al*. [35] and Patel [12] both show that younger people had more sensitivity to lockdowns while older people showed less negative effects than the rest of the age distribution. However, Ramiz *et al*. [21] show a U-shaped distribution of effect with both young and old being worse affected than the middle of the age distribution. Hale *et al*. [32] shows this effect is basically explicable as due to age-related differences in resilience and emotional stability.

Most international studies focus on 2020 presumably due to a lack of similar long lockdowns in peer countries after 2020 [1]. This limits the ability for some of the studies to provide evidence on the mental health implications of different government measures over time particularly with respect to the length and type of restrictions. Furthermore, some studies do not examine local restrictions but simply look at national policy variation [25–27]. Examining subnational policies is particularly important for Australia where there was significant variation in the restrictions throughout the pandemic for each Australian state and territory [5].

### Australia’s experience of COVID-19 lockdowns

There are a few key studies which aim to understand the effect of lockdowns on mental health in Australia – though most look only at 2020. All showed some negative effect of lockdowns on mental health even though the datasets and methods varied.

Butterworth *et al*. [36] analysed data from the longitudinal survey of Household, Income and Labour Dynamics in Australia (HILDA) to identify changes in the mental health of respondents from a pre-COVID-19 period to the COVID-19 period (between August and October 2020; with 10% of responses completed after October 2020 which was after the long Victorian lockdowns ended for 2020) using a representative sample. The timing of this study allowed researchers to investigate the changes between respondents in the state of Victoria who were exposed to stringent COVID-19 measures during the survey against other states, which had significantly less restrictions, using a difference-in-differences model. They found decreased mental health in both groups (Victoria and rest of Australia) from the pre pandemic to post pandemic period, and difference-in-differences estimation demonstrated a small but statistically significant mental health decline associated with the introduction of stay-at-home orders – particularly for those living in the state of Victoria who were experiencing stringent COVID-19 requirements for the second time. They also found the ‘lockdown’ effect was larger for women and in particular women in couples with children younger than 15 and women who lived in flats or apartments.

Fisher *et al*. [37] used a difference-in-differences approach to investigate if there was a difference in prevalence of symptoms of clinical depression and anxiety between Victoria and the rest of Australia. The study used survey data over two periods, April-May 2020 (during first Australian wide stay-at-home orders) and July-August 2020 (when all states and territories except Victoria had removed stay-at-home requirements). They gathered their own representative sample for the research. Fisher *et al*. found that there was no difference in the prevalence of anxiety and depression symptoms between Victoria and the rest of Australia during the first wave, but during the second wave there were substantial and statistically significantly higher rates of clinical depression and anxiety for Victorian respondents. Specifically, there was a doubling in prevalence of depression and anxiety symptoms over the period for Victorians compared to other jurisdictions where there were less stringent restrictions and symptoms of poor mental health remained stable to that measured in the first survey.

Goh *et al*. [38] collected survey data from across Australia using convenience sampling between May and December 2020. They lack a pre-lockdown baseline but instead show the change in outcomes as restrictions eased. They showed a decrease in depression and anxiety symptoms over time with younger people and people with caring responsibilities having particularly bad mental health during lockdown periods.

Klein *et al*. [39] collected repeated cross-sectional survey data before (January 2018) and during (September 2021) the pandemic in Australia. It is a largely representative sample though Canberra is oversampled. They look at a simple difference in means between those two waves and show that Melbournians had a higher jump in distress as measured by K10 scores than those elsewhere in Australia. Feeling the pandemic was being managed poorly and unwillingness to get a COVID-19 vaccine predicted higher distress.

Botha *et al*. [8] is particularly useful for understanding fine-grained evolution in mental health over the two years. The authors used daily, cross-sectional data collected from a large, convenience sample of Australian adults between May 2020 and December 2021 (574,306 adults in total over 611 days). They showed distress was higher during periods of lockdown, however, particularly interesting was what their intensive data allowed them to say about the dynamics of distress. There seems to be less effect for short lockdowns of pre-specified length, distress increased for longer lockdowns, and then after around ten weeks of lockdown distress plateaued or even decreased slightly. After the lifting of lockdowns, distress fell rapidly. In addition, in high lockdown jurisdictions (Victoria and NSW) there was evidence of a compounding effect with each additional lockdown being more distressing.

Finally, in the grey literature, there is a series of reports out of the Australian National University Centre for Social Research and Methods tracking a range of well-being indicators in data from ANU Poll, a representative, longitudinal survey. This includes distress across several waves of data collection during the pandemic period [40–42]. These papers track distress in a representative, longitudinal sample. They show that mean distress goes up and life satisfaction drops during lockdown periods and then recovers after the lockdowns are lifted. However, this is not a robust causal inference analysis, just reporting on polling trends over time.

This paper aims to understand how lockdowns affected distress during the entire period of 2020-2021. It does so by using the same representative, longitudinal, and frequent survey data that the ANU Poll reports used, but it uses a difference-in-differences approach for robust causal identification. This provides a more granular estimate for 2020 and the only estimate we are aware of that is this rigorous of the effects in 2021 anywhere in the world.

While the focus of this work is Australia, it also has broader implications. Australia was a very low-infection country, and its cities endured some of the longest lockdowns in the world [43]. This means we can separate the effect of disease from the effect of lockdowns better than in most of the rest of the world and we can look across a longer period of lockdown than other studies. This clarifies the effect of containment measures more broadly over a long period of time.

## Data and methods

### Data

#### The Oxford Covid Government Response Tracker Australian Subnational Dataset (OxCGRT).

For this study we draw on two sources of data. The first is the Oxford Covid Government Response Tracker Australian Subnational Dataset (OxCGRT) [1,5] which coded the policy settings of Australian Governments on every day between January 2020 and December 2021. This data contains an indicator that we take as our treatment variable C6 – Stay at home requirements. It codes 0 as no measures, 1 as recommended to stay at home, 2 as required to stay at home, and 3 as required to stay at home with minimal exceptions. We take any value of 2 or 3 as the jurisdiction being treated. During the two periods studied, lockdowns aligned well with the OxCGRT method of coding lockdowns, with a set value for metro and regional areas in each state and territory (except the Australian Capital Territory which did not have a regional code given the lack of substantial regional populations). Neither period of lockdown was targeted at a lower level, as in for example the lockdown of Sydney’s Northern Beaches for three weeks in late 2020 – early 2021. In one case, there was a localised, one week-long lockdown that ended on the first day of fieldwork for Wave 7 in South-Eastern Queensland. As this was not a sustained lockdown and was not in effect for the vast majority of the Wave, we choose to treat this as a control jurisdiction in 2021.

#### ANU Poll.

Other variables are drawn from the ANU Poll panel dataset. ANU Poll is conducted at varying intervals, although throughout the pandemic, it was carried out roughly once every three months. It is administered to the Social Research Centre’s Life In Australia panel; a nationally representative, probability-based panel that was first assembled in 2016. Panel members are recruited via phone (mobile and landline) with the survey is administered online. There were eight waves of data collected between April of 2020 and December of 2021 as shown in Table 1 (this excludes the larger group of ANU Poll waves that did not ask about psychological distress). The distribution of respondents by state is given in S1 Appendix. The data is released by wave so datasets and documentation on each wave (including a technical report) can be found at the ANU Poll Dataverse which has entries for each wave. Note that the data used here is only available with an application to the Australia Data Archive. This analysis uses a longitudinal dataset of these waves [44].

**Table 1:** Waves of ANU Poll collected during 2020 and 2021 (excluding waves without a measure of psychological distress).

Wave	Survey window	Sample size	Per cent of January 2020 survey responded
1 – April 2020	14th to 27th April, 2020	3,155	88.8
2 – May 2020	11th to 25th May, 2020	3,249	91.0
3 – August 2020	10th to 24th August, 2020	3,061	85.9
4 – November 2020	9th to 23rd November, 2020	3,029	84.9
5 – January 2021	18th January to 1st February, 2021	3,459	83.8
6 – April 2021	12th to 26th April, 2021	3,286	80.8
7 – August 2021	10th to 23rd August, 2021	3,135	71.1
8 – October 2021	12th to 26th October, 2021	3,474	68.6

There are also separate technical reports for each Life in Australia wave (of which ANU Poll is a subset) which can be found online, but are not collected in a repository. The best single source for information on the Life in Australia panel is the methodological report on the building of the panel [45].

While there were waves before April 2020, none of these had the outcome variable needed to be included in this study. The Life in Australia wave numbers and ANU Poll wave numbers are different to the wave numbers used here. The reason for this is that psychological distress was not a part of the core module of ANU Poll previously and by the time it was included in order to measure distress caused by the pandemic and the response to the pandemic, it was too late to measure a baseline level. However, there was a measure taken in 2017 as part of an earlier wave focused on mental health and we draw on this data, as well as data from other samples that can help to establish parallel trends for the 2020 Period. This analysis is detailed in S2 Appendix.

Psychological distress was measured by the Kessler 6 (K6) scale. The K6 comprises six items and has been widely used and validated in many epidemiological studies [46]. Respondents who score highly on this measure are considered to be at risk of a serious mental illness (other than a substance use disorder). The measure asks respondents to recall their feelings over the past four weeks. This may then not be a good picture of their feelings at that particular moment and its particular policy settings. The policy settings are relatively stable, but the reader should bear in mind there are some jurisdictions in some waves where there have been policy changes within that 30 day period. This means earlier feelings from a different policy setting might inform responses, for example, Victoria and ACT in Wave 7.

The K6 measure of psychological distress used in this paper has been constructed to have a minimum value of 6 and a maximum value of 30. In February 2017 when the question was last asked, the average value was 11.2. By April 2020, the score had increased to have a mean of 11.9, and a standard deviation of 4.8.

**Fig 1:**
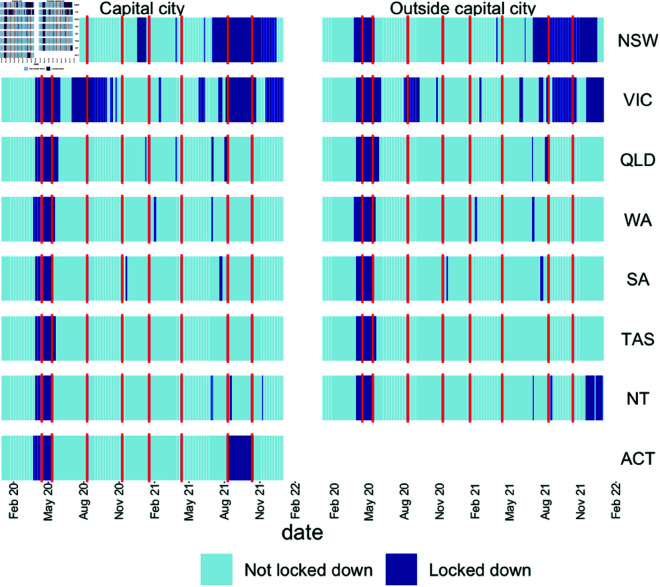
Lockdown status by wave by area. NB: There was no separate coding for the ACT outside of Canberra.

The lockdown status by ANU Poll wave is shown in Fig 1. There are clearly two periods of sustained lockdown that are captured by the data. There are also other periods of lockdown during the study period, these were generally short and so occurred between waves of data collections and were not considered in this study.

#### Exploring patterns in the dependent variable.

**Fig 2:**
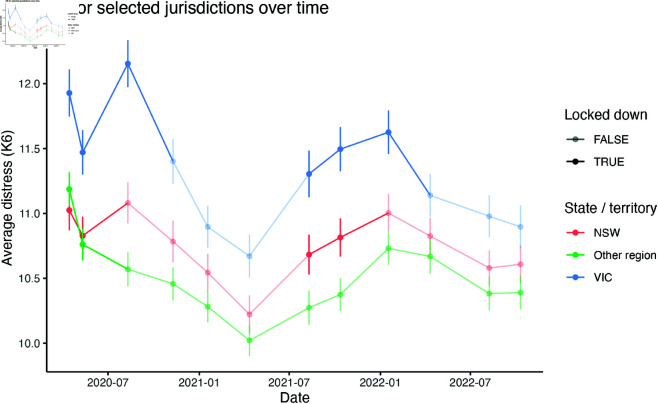
Distribution of distress over time for NSW, Victoria and other regions. Locked down periods are those where any area in the region is under a stay-at-home order. Later waves after the removal of lockdowns are provided for context. While the ACT had separate treatment timing, it is excluded for clarity due to wide error bars and noisy trends.

Fig 2 below presents the trends over time in distress by jurisdiction. The reason for this grouping is that New South Wales and Victoria had distinct periods of extended lockdown that the other jurisdictions lacked. Note that ACT policy was independent enough of the other two that it did not make sense to group it in with either but the sample size here is small enough that data were very noisy, and the standard errors were quite wide. For this reason, we exclude it from the figure to improve clarity.

Fig 2 shows several clear patterns. First, there is a substantial increase in distress in Victoria during the first lockdown period, a lesser jump up in NSW and a decrease in distress in the same period in other regions (where many jurisdictions had left lockdown already by the May 2020 wave). In the second period of extended lockdowns in mid to late-2021 we do not see such a divergence in levels of psychological distress between jurisdictions. Instead, the series largely have the same trend in distress levels regardless of treatment status. Distress was increasing on average for everyone.

There are some limits in what can be interpreted from Fig 2 however. Mean levels of distress are grouped as though everyone living in a state has a jurisdiction wide, binary treatment however we know that major cities had much more stringent lockdown policies than in regional areas of the most affected states (NSW and Victoria).

### Empirical approach

In order to study the effect of lockdowns and try to isolate a causal effect, we use the Callaway-Sant’Anna (CS) staggered difference-in-differences approach [7]. All estimates are made using the did R package [47] taking the never treated group as a control group. The CS approach is based on estimating two-period average treatment effects on the treated (ATTs) for each group *g* (cohort of units entering treatment at a given time) and time *t* and then aggregating them in ways that make sense analytically. This allows for the estimation of an overall ATT. However, for this study we use more granular estimates of a group-time average treatment effect on the treated (formally *ATT*(*g*,*t*) but we will also refer to it less formally as the GTATT).

We use the CS approach along with the package’s default tilting-based doubly robust estimator [48] to estimate $ATT_{dr}^{nev}(g,t)$. This is the ATT with a never-treated comparison group (as opposed to not-yet-treated).

We include the following covariates: gender, age, indigenous status, immigrant status, speaking a language other than English, highest education level, SEIFA quintile and living in a capital city or not as possible confounders which could have a time-varying effect on outcomes. Models designed to test treatment effect heterogeneity use the same specification but for specific subsets of the sample.

Because each period is somewhat idiosyncratic (e.g. policies in place are qualitatively different, lockdown has lasted different amounts of time) we focus on disaggregated estimates for each group and time. Although we also report an overall ATT, the individual GTATTs are just as important for understanding what is going on.

There are two main assumptions to identify the GTATT with difference-in-difference. The first assumption is that the population did not anticipate the introduction of lockdowns as these were generally introduced quickly after an outbreak (which was by definition unexpected under zero-COVID policy) [5,6]. In many cases lockdowns were announced with just a few hours’ notice in order to stop people from changing their behaviour in harmful ways between announcement and an order going into effect. The second assumption – parallel trends – is less straight forward in either period. In The 2020 Period we lack enough pre-treatment outcomes data to properly test for parallel trends. Because of this we rely on supplementary data to help argue for parallel trends in the 2020 Period in S2 Appendix. Also, because all lockdowns in the 2020 Period start in the same wave, in this analysis the treatment effect estimated is the lifting of lockdowns (as this was staggered) rather than the imposition of lockdowns. Assuming distress bounces back somewhat after lifting as seems credible from other research [8,19], this can tell us something about the effect of being in lockdown.

While the canonical Callaway-Sant’Anna [7] approach assumes no treatment prior to the start of the study (a condition we do not meet in the 2021 Period), this does not actually have to be the case. So long as the effect of the prior treatment has ‘washed out’ by the time of the 2021 Period or at least the effect is no longer time-varying (and therefore will be differenced out in the fixed effects) the estimate will not be biased. This is particularly important because treatment between the two periods is correlated (Victoria and NSW tend to have longer lockdowns in both periods). Fortunately, this is a somewhat testable assumption as we can graph the difference in outcomes over time for the control and treatment groups as in Fig 1 and see if they have settled back into parallel trends by mid-2021. We can also look at the GTATT plots as event studies disaggregated into groups in order to check treatment effects for the pre-treatment period are zero. Finally, this assumption seems plausible given the dynamics tracked by Botha *et al*. [8] who showed a relatively rapid wash-out in their daily surveying of Australians over the course of the pandemic.

Code for this analysis is available at https://github.com/pbrehill/COVID-psych-distress/.

## Results

### The 2020 period

In the Callaway-Sant’Anna staggered difference-in-differences approach it is standard to estimate treatment effects not for individual units but for groups that all entered treatment in the same wave. These are generally referred to by reference to first treatment wave. In The 2020 Period we have two groups, Group 2 (South Australia (SA), Northern Territory (NT) and the Australian Capital Territory (ACT)) and Group 3 (NSW, Queensland (QLD) and Tasmania (TAS)). Victoria is the never-treated comparison group.

In the 2020 Period we study the effect of imposing lockdown, so a negative treatment effect means a decrease in distress once lockdown is lifted. Table 2 shows the results of the analysis for the 2020 Period both in terms of K6 units (a scale of 6 – 30) and standard deviations of the K6 scale. There is no statistically significant treatment effect at time 2 for either group, both Group 3 which is still in treatment and Group 2 which is not. At time 3 we see a statistically significant effect in Group 3 (a negative effect being a fall in distress) of -0.580 or -0.121 standard deviations. There is no statistically significant effect in Group 2, perhaps due to small sample size making this group underpowered for estimating a relatively small effect. It is not possible to establish if there is a lingering treatment effect beyond these two periods because Victoria then left lockdown in the next wave meaning there was no longer a never-treated group for comparison.

**Table 2:** Group-time average treatment effects on the treated for the 2020 Period.

Group	Time	Period	Lockdown lifted	ATT g,t in K6 units	ATT g,t in SD units
2. ACT, NT, WA	2	May 2020	Yes	0.241	0.050
				(0.649)	(0.136)
	3	August 2020	Yes	-0.792	-0.166
				(0.853)	(0.178)
3. NSW, SA, QLD, TAS	2	May 2020	No	0.067	0.014
				(0.131)	(0.027)
	3	August 2020	Yes	-0.580	-0.121
				(0.105)	(0.021)
Overall ATT				-0.478	-0.100
				(0.088)	(-0.019)

The ATT here is composed of a weighted average across the groups and treatment periods per Callaway and Sant’Anna [7]. It is -0.478 on the K6 scale or -0.100 units on a standard deviation scale. This is statistically significant at α=0.95.

### The 2021 period

In The 2021 Period we study the effect of imposing lockdown, so a positive treatment effect means a rise in distress once lockdown is introduced. We have the benefit of a longer period of data pre-treatment. The treatment effects for each period are shown in Table 3. We can see the pre-treatment periods suggest that there is no time-variant difference in the treatment and control groups before the treatment is introduced. However, there does not appear to be any effect after the treatment is introduced either. No GTATTs are statistically significant at α=0.95.

**Table 3:** Group-time average treatment effects on the treated for the 2021 Period.

Group	Time	Period	Lockdown in effect	ATT g,t in K6 units	ATT g,t in SD units
NSW and metro VIC	5	January 2021	No	-0.266	-0.056
				(0.299)	(0.063)
	6	April 2021	No	0.092	0.019
				(0.418)	(0.087)
	7	August 2021	Yes	0.174	0.036
				(0.527)	(0.110)
	8	October 2021	Yes	0.074	0.015
				(0.607)	(0.127)
Regional VIC and ACT	5	January 2021	No	-0.310	-0.065
				(1.015)	(0.212)
	6	April 2021	No	0.689	0.144
				(2.113)	(0.442)
	7	August 2021	No	-0.286	-0.060
				(2.481)	(0.519)
	8	October 2021	Yes	0.230	0.048
				(0.664)	(0.139)
ATT				0.156	0.033
				(0.521)	(0.109)

The ATT point estimate here is 0.156 points on the K6 scale or 0.033 points on a standard deviation scale. This is not a statistically significant effect.

## Discussion

In the 2020 Period, we find a statistically significant drop in distress for Group 3 (the large group made up of NSW, SA, QLD, and TAS) when lockdowns are lifted. The ATT is -0.580 points. This effect is relatively small, it amounts to just -0.121 standard deviations in the K6 pre-treatment but it is roughly in-line with the effect found by Butterworth *et al*. [36] (who studied the effect of imposing rather than lifting lockdown). If we are to assume this is a bouncing back to pre-lockdown levels of distress (which seems reasonable given Botha *et al*.’s [8] findings), this implies a negative effect on distress from entering lockdown. We do not see a statistically significant effect of leaving lockdown on the other jurisdictions from Group 2 (WA, NT, ACT). If the true effect for this smaller group were similar in magnitude to that estimated for Group 3, the sample would be underpowered to measure this effect.

Turning to the 2021 Period, the results are more unexpected. There is no measurable effect in either group, in either period. The first possible justification is that the lockdowns simply did not have an effect in their own right. It is possible that people became more acclimatised to life in lockdown after already having gone through lockdowns before ala Fancourt *et al*. [19]. This is in line with the peaks of distress in 2021 being lower even after a longer time in lockdown. This kind of behaviour is adaptation to cumulative stress where, through a variety of possible mechanisms, the effect of a stressor on an individual’s wellbeing is reduced over time [49–51]. Another (not mutually incompatible) possibility is that vaccinations in and before the 2021 Period may have ameliorated the effect of lockdowns on distress as there was a clear end in sight – lockdowns will be removed once vaccination milestones had been reached [5]. For those who were vaccinated, distress about getting the virus may have been lowered as well [52]. This would be in line with Klein *et al*.’s [39] findings that lower willingness to vaccinate and trust in public health authorities predict higher distress during this period. The effect we measure here is not in line with Botha *et al*.’s [8] findings researching Australians in the same timeframe. Their paper shows there was a reasonable uptick in depression and a smaller uptick in anxiety during the 2021 lockdowns. This is perhaps due to a difference in outcomes, different sampling practices (theirs is a convenience sample) or simply our less granular time resolution.

A similar, but distinct explanation is that the effect may have been small and that changes in control jurisdictions at the same time obscured the negative effects of the lockdown policies. This is supported by the fact that during the 2021 Period individuals in both treatment and control jurisdictions have increased distress. Control jurisdictions were experiencing their first serious community spread of the virus while treatment jurisdictions were more used to community spread, and the containment measures in place may have lowered anxiety around this spread further [5]. Control jurisdictions also had lower vaccination rates, potentially fuelling distress around risk of disease or distress from the unvaccinated about living under additional restrictions (for example a ban on air travel, bans on doing some jobs) [53].

This complexity around understanding the exact mechanism behind this relationship between lockdowns and distress is discussed more widely in S4 Appendix. It discusses the fact that lockdowns, increase in case numbers and increases in anxiety about the virus are all highly correlated.

## Conclusion

While there appears to have been a negative effect of COVID-19 lockdowns on psychological distress in Australia during the early part of the pandemic (March – October 2020) as measured by the lifting of lockdown, we did not estimate any statistically significant increase in distress from imposing lockdowns for the period of extended lockdowns in 2021 (June – November 2021). In the 2020 Period, we see a statistically measurable effect of leaving lockdown with an ATT of -0.100 standard deviations (that is a reduction in distress).

The story is more complicated for the 2021 Period. Distress did go up in locked-down jurisdictions, but it increased similarly in other jurisdictions as well. There was no measurable effect in any of the group-time estimates or in the overall average. It seems that if lockdowns increased psychological distress in the 2021 Period, other developments in the pandemic in control jurisdictions caused a corresponding increase. The rolling out of vaccines which proceeded faster in locked down jurisdictions (for reasons both of greater supply and demand), the spread of cases in control jurisdictions in this 2021 Period (in contrast to the first where there was no spread in control jurisdictions), potential adaptation to lockdowns, and the different goals of the lockdowns (containing spread until reaching widespread vaccination rather than eradicating the virus) made this second lockdown period categorically different from the first. While distress increased in locked down jurisdictions, it did not seem like this increase was measurably greater than it would have been counterfactually.

These lockdowns represent some of the longest (in terms of total days spent in lockdown) in the world, while the number of cases in Australia during almost the entire study period was extremely low by global standards. This creates a relatively clean natural experiment on the effect of containment measures on distress. These findings then are not just useful for understanding the effect of COVID-19 lockdowns but may be useful in weighing the use of containment policies if they might be required in future emergencies.

## Supporting information

S1 Appendix Geographic distribution of all ANU Poll unique respondents during the period studied by state/territory. (DOCX)

S2 Appendix Testing parallel trends in the 2020 Period of the study with supplemental data. (DOCX)

S3 Appendix Heterogeneity analysis. (DOCX)

S4 Appendix Remarks on separating out the effects of correlated treatments. (DOCX)
