# Supplementary material for: Understanding the effect of stay-at-home orders on psychological distress during the COVID-19 pandemic: Evidence from a longitudinal study in Australia
Source: PLoS One. 2025 Jul 2;20(7):e0325753. doi: 10.1371/journal.pone.0325753 (PMC12221174; doi:10.1371/journal.pone.0325753)
Supplement: S1 Appendix — (DOCX) [file pone.0325753.s001.docx]

# S1 Appendix - Geographic distribution of ANU Poll respondents

The distribution of respondents by area is shown in Error: Reference source not found for all unique respondents during the period totalling 6623 individuals.

| Jurisdiction | Unique respondents |
| --- | --- |
| ACT | 167 |
| Sydney | 1291 |
| Rest of NSW | 804 |
| Darwin | 28 |
| Rest of NT | 15 |
| Brisbane | 672 |
| Rest of Queensland | 580 |
| Adelaide | 523 |
| Rest of SA | 120 |
| Hobart | 86 |
| Rest of TAS | 87 |
| Melbourne | 1288 |
| Rest of VIC | 431 |
| Perth | 438 |
| Rest of WA | 93 |
