# Supplementary material for: Understanding the effect of stay-at-home orders on psychological distress during the COVID-19 pandemic: Evidence from a longitudinal study in Australia
Source: PLoS One. 2025 Jul 2;20(7):e0325753. doi: 10.1371/journal.pone.0325753 (PMC12221174; doi:10.1371/journal.pone.0325753)
Supplement: S2 Appendix — (DOCX) [file pone.0325753.s002.docx]

# S2 Appendix - Testing parallel trends in the 2020 Period of the study with supplemental data

The only ANUpoll data we have on pre-treatment levels of distress is unfortunately from a 2017 wave. Of course, one datapoint before lockdown is not enough to measure a trend, but we can also learn something from the trend between the 2017 wave and April 2020 because the policies have not diverged between jurisdictions yet, even though a lockdown entered place in all of them. In this way the treatment jurisdictions have not been different from the control jurisdictions because they have all been exposed to the same policies. To the extent this is informative, there seems to have been a parallel trend across the period between NSW and other jurisdictions. Victoria however is not suggestive of such a pattern. Distress jumps substantially between the 2017 measurement and April 2020 suggesting one of three things: either there is no parallel trend in the pre-lockdown period, and something had already raised Victoria’s distress, the lockdowns in Victoria in early 2020 caused much greater distress in Victoria than in other jurisdictions, or there was an earlier divergence that is no longer relevant by 2020. To figure out whether this data points to a parallel trends violation we need to turn to other data.


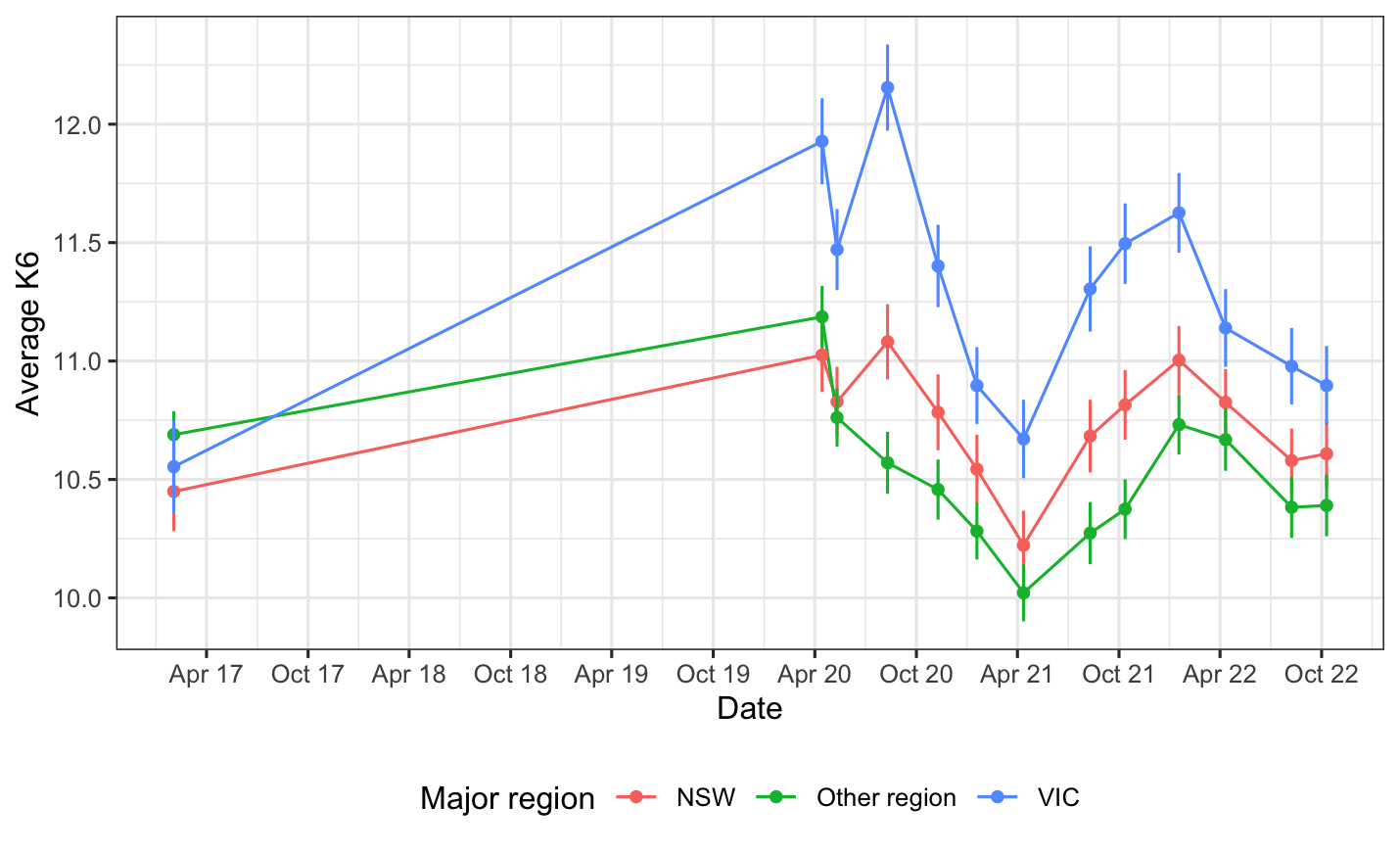


K6 across time in ANUpoll.

We can also boost the credibility of the parallel trends assumption for the 2020 Period by turning to other data sources sampling from the same population. The Household Income and Labour Dynamics in Australia (HILDA) survey is collected once per year and gathers two measures that can be useful proxies of K6, the MHI-5, and the K10 (44). The former is a positive measure of mental health (and is the outcome used by Butterworth et al. (36)), the latter is a measure of psychological distress measuring the same construct as the K6 but with more items. While K10 is more directly comparable, it is unfortunately only collected bi-yearly in HILDA while the MHI-5 items are collected yearly. There are therefore reasons we might want to look at parallel trends in both, K10 having greater conceptual relevance and MHI-5 giving better resolution over time. The MHI-5 plots the two variables going back to 2012 for NSW, Victoria and control jurisdictions (which for the 2020 Period includes the ACT) in 2020 across both K10 and MHI-5. It shows largely parallel trends back to 2013. This might indicate that there are likely (latent) parallel trends in the ANUpoll sample as well and that the strange change between 2018 and 2020 is due to noise rather than some underlying experience the Victorian population had.


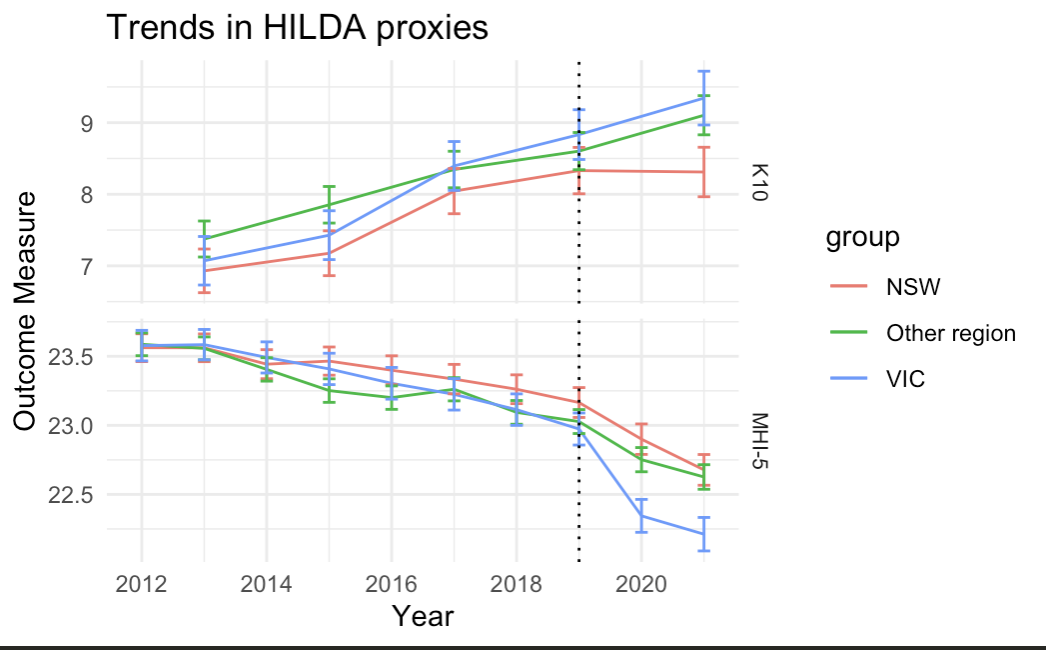


Trends in HILDA proxies for K6. (dashed line shows the point at which parallel trends would diverge due to treatment).
