# Supplementary material for: Understanding the effect of stay-at-home orders on psychological distress during the COVID-19 pandemic: Evidence from a longitudinal study in Australia
Source: PLoS One. 2025 Jul 2;20(7):e0325753. doi: 10.1371/journal.pone.0325753 (PMC12221174; doi:10.1371/journal.pone.0325753)
Supplement: S3 Appendix — (DOCX) [file pone.0325753.s003.docx]

# S3 Appendix - Heterogeneity analysis

To find treatment effect heterogeneity individual models were fit for different subgroups. We then Z-test the difference between the treatment effect estimate from a base case per Clogg et al. (1995) as

$$Z=\frac{\beta_{1}-\beta_{2}}{\sqrt{\left( SE_{\beta_{1}} \right)^{2}+\left( SE_{\beta_{2}} \right)^{2}}}.$$

The table below presents group-time average treatment effects for the different groupings in the 2020 Period. Note that the sample sizes tend to be quite small here and it is likely that if there is any true effect, they are underpowered for measuring that effect. For the sake of simplicity, we only examine the first period after treatment for treatment effect heterogeneity.

Overall, there is no measurable heterogeneity according to any of these characteristics aside from having an undergraduate degree in Group 5. Where distress was -1.090 points lower than those who had only finished Year 12 and this difference was statistically significant at $\alpha= 0.05$.

Results for models testing treatment effect heterogeneity in the 2020 Period

| Group | Variable family | Sample | Difference | Z | p | n |
| --- | --- | --- | --- | --- | --- | --- |
| ACT, NT, WA | Language | English is first language (base case) |  |  |  | 382 |
|  |  | LOTE is first language | -0.057 | -0.088 | 0.930 | 52 |
|  | Age | Age 18 - 24 |  |  |  | 11 |
|  |  | Age 25 - 34 | 0.764 | 0.324 | 0.746 | 48 |
|  |  | Age 35 – 44 (base case) |  |  |  | 72 |
|  |  | Age 45 - 54 | 0.756 | 0.379 | 0.705 | 72 |
|  |  | Age 55 - 64 | 0.381 | 0.276 | 0.782 | 80 |
|  |  | Age 65 - 74 | 0.333 | 0.211 | 0.833 | 93 |
|  |  | Age 75+ | 0.159 | 0.129 | 0.897 | 57 |
|  | Education | Less than Year 12 | -0.102 | -0.033 | 0.974 | 41 |
|  |  | Year 12 (base case) |  |  |  | 82 |
|  |  | Certificate 3 or higher | -0.282 | -0.156 | 0.876 | 109 |
|  |  | Undergraduate degree | 0.173 | 0.095 | 0.924 | 143 |
|  |  | Postgraduate degree | -0.897 | -0.369 | 0.712 | 58 |
|  | Socio-economic status | SEIFA quartile 2 | -0.170 | -0.055 | 0.956 | 28 |
|  |  | SEIFA quartile 2 | 0.005 | 0.002 | 0.998 | 61 |
|  |  | SEIFA quartile 3 (base case) |  |  |  | 77 |
|  |  | SEIFA quartile 4 | -0.372 | -0.179 | 0.858 | 110 |
|  |  | SEIFA quartile 5 | -0.592 | -0.264 | 0.792 | 157 |
|  | Country of birth | Born in Australia (base case) |  |  |  | 280 |
|  |  | Born in English-speaking country | -0.213 | -0.187 | 0.852 | 92 |
|  |  | Born in non-English speaking country | -0.029 | -0.012 | 0.990 | 61 |
|  | Gender | Female (base case) |  |  |  | 242 |
|  |  | Male | -0.062 | -0.068 | 0.946 | 190 |
| NSW, SA, QLD, TAS | Language | English is first language (base case) |  |  |  | 1596 |
|  |  | LOTE is first language | -0.150 | -0.203 | 0.839 | 249 |
|  | Age | Age 18 - 24 | 2.218 | 2.220 | 0.026 | 45 |
|  |  | Age 25 - 34 | 0.674 | 0.791 | 0.429 | 203 |
|  |  | Age 35 – 44 (base case) |  |  |  | 279 |
|  |  | Age 45 - 54 | 0.757 | 1.123 | 0.261 | 288 |
|  |  | Age 55 - 64 | 0.781 | 1.160 | 0.246 | 390 |
|  |  | Age 65 - 74 | 1.214 | 1.785 | 0.074 | 417 |
|  |  | Age 75+ | 0.416 | 0.594 | 0.553 | 223 |
|  | Education | Less than Year 12 | -0.173 | -0.274 | 0.784 | 254 |
|  |  | Year 12 (base case) |  |  |  | 269 |
|  |  | Certificate 3 or higher | -0.521 | -0.735 | 0.462 | 519 |
|  |  | Undergraduate degree | -1.090 | -2.843 | 0.004 | 533 |
|  |  | Postgraduate degree | -0.434 | -1.108 | 0.268 | 271 |
|  | Socio-economic status | SEIFA quartile 1 | -0.602 | -1.063 | 0.288 | 373 |
|  |  | SEIFA quartile 2 | 0.708 | 0.884 | 0.377 | 367 |
|  |  | SEIFA quartile 3 (base case) |  |  |  | 401 |
|  |  | SEIFA quartile 4 | -0.072 | -0.149 | 0.882 | 314 |
|  |  | SEIFA quartile 5 | -0.335 | -0.565 | 0.572 | 388 |
|  | Country of birth | Born in Australia (base case) |  |  |  | 1378 |
|  |  | Born in English-speaking country | -0.243 | -0.348 | 0.728 | 226 |
|  |  | Born in non-English speaking country | 0.326 | 0.420 | 0.674 | 236 |
|  | Gender | Female (base case) |  |  |  | 1025 |
|  |  | Male | 0.147 | 0.223 | 0.823 | 819 |

*Base case characteristic
NB: The indigenous sample of four for this group is not reported as the sample is too small for the estimate to be reliable. The 18–24-year-old sample for ACT, WA and NT was not reported for similar reasons.

We for the most part did not find heterogeneity in the treatment effects. The only statistically significant drivers of heterogeneity were in the 2020 Period where those with an undergraduate degree in Group 3 had substantially lower distress than those who had just finished Year 12 (1.090 points less). This is in-line with existing research showing that those with higher levels of education had lower distress around the world. This was perhaps due to greater flexibility to work from home in professional jobs, correspondingly low levels of job loss and greater financial security.

The other statistically significant driver was being 18 – 24 in Group 3 as this group has substantially higher outcomes than the base case. This may be because younger people suffered more from lockdowns. It may also be an issue with the very small sample size for this young cohort. The test used here which relies on the central limit theorem may simply be inappropriate.

We tested heterogeneity for the 2021 Period the same way as in the 2020 Period. Again, there was little in the way of measurable heterogeneity. In this case there were in fact no statistically significant effects at $\alpha= 0.05$.

Table 5: Results for models testing treatment effect heterogeneity in the 2021 Period

| Group | Variable family | Sample | Difference | Z | p | n |
| --- | --- | --- | --- | --- | --- | --- |
| NSW | Language | English is first language* |  |  |  | 736 |
|  |  | LOTE is first language | 0.281 | 0.308 | 0.758 | 132 |
|  | Age | Age 18 - 24 | 3.316 | 1.674 | 0.094 | 34 |
|  |  | Age 25 - 34 | -0.122 | -0.094 | 0.925 | 127 |
|  |  | Age 35 – 44* |  |  |  | 150 |
|  |  | Age 45 - 54 | 0.194 | 0.135 | 0.893 | 152 |
|  |  | Age 55 - 64 | -0.184 | -0.133 | 0.894 | 201 |
|  |  | Age 65 - 74 | -0.157 | -0.074 | 0.941 | 218 |
|  |  | Age 75+ | -1.142 | -0.988 | 0.323 | 106 |
|  | Education | Less than Year 12 | -0.098 | -0.136 | 0.892 | 108 |
|  |  | Year 12* |  |  |  | 96 |
|  |  | Certificate 3 or higher | -0.848 | -0.560 | 0.576 | 238 |
|  |  | Undergraduate degree | -0.545 | -0.390 | 0.697 | 262 |
|  |  | Postgraduate degree | -0.915 | -0.367 | 0.714 | 131 |
|  | Socio-economic status | SEIFA quartile 1 | -0.732 | -0.918 | 0.359 | 158 |
|  |  | SEIFA quartile 2 | -0.327 | -0.604 | 0.546 | 182 |
|  |  | SEIFA quartile 3* |  |  |  | 193 |
|  |  | SEIFA quartile 4 | -0.527 | -0.868 | 0.386 | 102 |
|  |  | SEIFA quartile 5 | -0.104 | -0.151 | 0.880 | 232 |
|  | Country of birth | Born in Australia* |  |  |  | 649 |
|  |  | Born in English-speaking country | 0.437 | 0.856 | 0.392 | 103 |
|  |  | Born in non-English speaking country | 0.541 | 0.735 | 0.462 | 111 |
|  | Gender | Female* |  |  |  | 484 |
|  |  | Male | 0.217 | 0.271 | 0.786 | 384 |
| VIC, ACT | Language | English is first language |  |  |  | 770 |
|  |  | LOTE is first language | -0.675 | -0.120 | 0.905 | 188 |
|  | Age | Age 18 - 24 | 3.267 | 0.870 | 0.385 | 46 |
|  |  | Age 25 - 34 | 0.671 | 0.345 | 0.730 | 132 |
|  |  | Age 35 – 44* |  |  |  | 154 |
|  |  | Age 45 - 54 | 0.855 | 0.262 | 0.793 | 148 |
|  |  | Age 55 - 64 | 1.048 | 0.559 | 0.576 | 176 |
|  |  | Age 65 - 74 | 1.043 | 0.392 | 0.695 | 167 |
|  |  | Age 75+ | 2.129 | 1.137 | 0.255 | 110 |
|  | Education | Less than Year 12 | 0.250 | 0.304 | 0.761 | 108 |
|  |  | Year 12* |  |  |  | 116 |
|  |  | Certificate 3 or higher | 1.031 | 0.557 | 0.578 | 224 |
|  |  | Undergraduate degree | 0.329 | 0.181 | 0.856 | 246 |
|  |  | Postgraduate degree | 1.461 | 0.624 | 0.533 | 158 |
|  | Socio-economic status | SEIFA quartile 1 | 1.123 | 1.094 | 0.274 | 137 |
|  |  | SEIFA quartile 2 | -0.171 | -0.313 | 0.754 | 148 |
|  |  | SEIFA quartile 3* |  |  |  | 143 |
|  |  | SEIFA quartile 4 | 1.182 | 1.436 | 0.151 | 227 |
|  |  | SEIFA quartile 5 | 1.538 | 1.553 | 0.121 | 325 |
|  | Country of birth | Born in Australia* |  |  |  | 662 |
|  |  | Born in English-speaking country | 0.398 | 0.727 | 0.467 | 92 |
|  |  | Born in non-English speaking country | -1.575 | -0.386 | 0.699 | 194 |
|  | Gender | Female* |  |  |  | 540 |
|  |  | Male | 0.420 | 0.558 | 0.577 | 433 |

As with the previous period, there was no treatment effect heterogeneity measured in this analysis.
