# Supplementary material for: Understanding the effect of stay-at-home orders on psychological distress during the COVID-19 pandemic: Evidence from a longitudinal study in Australia
Source: PLoS One. 2025 Jul 2;20(7):e0325753. doi: 10.1371/journal.pone.0325753 (PMC12221174; doi:10.1371/journal.pone.0325753)
Supplement: S4 Appendix — (DOCX) [file pone.0325753.s004.docx]

# S4 Appendix – Remarks on separating out the effects of correlated treatments

One theoretical issue that is worth discussing is the difficulty in disentangling different causal factors because several factors that theoretically influence distress are highly correlated in time. The policy responses we are interested in studying were not exogenous shocks, lockdowns were only introduced where there was a clear need for them (where cases were rising) and alongside other policy interventions. In addition, there may be an interference effect as lockdowns in one jurisdiction could cause anxiety in other jurisdictions about the risk of further spread.

While there is no easy solution to these questions, we propose an approach that mitigates these criticisms as much as possible. First and foremost, to the extent there are particulars of certain jurisdictions at certain times – different case numbers, different sets of containment measures already in place, different levels of COVID anxiety. To address these issues we report group-time and time average treatment effects on the treated for context. We also average these up into a total ATT for the period though this should be interpreted in the context of the individual group-time estimates that make it up.

On the correlation of treatment with cases, we define a specific counterfactual for causal inference – that the lockdown was not introduced but that cases also stayed on the trajectory they were on in the control jurisdictions. This means it is not strictly the case that the counterfactual would have been attainable through a policy choice as in many difference-in-differences designs. However, this does not affect the validity of the design, only the way in which we interpret results. While we are interested in distress caused by lockdowns, it would be wrong to interpret this as avoidable. Our aim is just to see what the cost of lockdowns was, one could of course (outside the scope of this paper) argue that this cost was or was not worth the benefits in containing the virus.

On the problem of correlated interventions (for example stay at home orders being correlated with other containment indicators), we offer three solutions. Firstly, we take a somewhat broader definition of lockdown. We are not strictly interested just in the effect of stay-at-home orders to the exclusion of other policy responses. Instead, lockdowns were part of a raft of other policy interventions that often happened at the same time, school closures, the cancelling of events and closure of state borders. The effect should be interpreted not as an effect of stay-at-home orders specifically (though we operationalise the treatment with stay-at-home orders) but instead from this stack of treatments that coincided with stay-at-home orders. Secondly, our choice of methods means that while there will be other containment indicators that could not be seen as part of a lockdown that are different across treatment and control jurisdictions before treatment begins (for example, limits on the number of guests in a home), these will not have a biasing effect so long as the impact of these is not time-varying at the point where the lockdown is introduced.

Finally, there is the issue of dealing with the anxiety caused by COVID-19 cases both in the locked down state and in other jurisdictions. Within jurisdictions that are locked down, to some extent we cannot separate out anxiety from the signalling effect of the lockdown because they are so intrinsically linked. In fact, raising the salience of the pandemic in people’s minds is part of the mechanism by which lockdowns work. The laws are supposed to have a signalling effect that regulates individual behaviour beyond the precise letter of the law and importantly, which helps to ensure people follow restrictions. For this reason, we do not seek to separate out the effect within the treated jurisdiction, however, we still must worry about the interference effect in other jurisdictions. This is a legitimate concern and one that is tough to address methodologically. Here we rely on the disaggregation of treatment effects to at the very least provide the opportunity to discuss the extent to which we would expect estimates to be biased by interference.
